# Applying Computer Adaptive Testing to Optimize Online Assessment of Suicidal Behavior: A Simulation Study

**DOI:** 10.2196/jmir.3511

**Published:** 2014-09-11

**Authors:** Derek Paul De Beurs, Anton LM de Vries, Marieke H de Groot, Jos de Keijser, Ad JFM Kerkhof

**Affiliations:** ^1^Faculty of Psychology and EducationDepartment of Clinical PsychologyVU University AmsterdamAmsterdamNetherlands; ^2^EMGO Institute for Health and Care ResearchAmsterdamNetherlands; ^3^Perziq.comAmsterdamNetherlands; ^4^GGZ Foundation for Mental Health Care Friesland and Groningen UniversityGroningenNetherlands

**Keywords:** suicide, psychometrics, computing methodologies, Internet, suicidal ideation, risk assessment

## Abstract

**Background:**

The Internet is used increasingly for both suicide research and prevention. To optimize online assessment of suicidal patients, there is a need for short, good-quality tools to assess elevated risk of future suicidal behavior. Computer adaptive testing (CAT) can be used to reduce response burden and improve accuracy, and make the available pencil-and-paper tools more appropriate for online administration.

**Objective:**

The aim was to test whether an item response–based computer adaptive simulation can be used to reduce the length of the Beck Scale for Suicide Ideation (BSS).

**Methods:**

The data used for our simulation was obtained from a large multicenter trial from The Netherlands: the Professionals in Training to STOP suicide (PITSTOP suicide) study. We applied a principal components analysis (PCA), confirmatory factor analysis (CFA), a graded response model (GRM), and simulated a CAT.

**Results:**

The scores of 505 patients were analyzed. Psychometric analyses showed the questionnaire to be unidimensional with good internal consistency. The computer adaptive simulation showed that for the estimation of elevation of risk of future suicidal behavior 4 items (instead of the full 19) were sufficient, on average.

**Conclusions:**

This study demonstrated that CAT can be applied successfully to reduce the length of the Dutch version of the BSS. We argue that the use of CAT can improve the accuracy and the response burden when assessing the risk of future suicidal behavior online. Because CAT can be daunting for clinicians and applied scientists, we offer a concrete example of our computer adaptive simulation of the Dutch version of the BSS at the end of the paper.

## Introduction

### Background

Suicide ideation is defined as the presence of thoughts, plans, and wishes in an individual to end his/her own life [1]. Assessment of suicide ideation is argued to be important because it may precede an attempt and it could provide information on the seriousness and lethality of the suicidal intention [2]. The Beck Scale for Suicide Ideation (BSS) is the 19-item self-report version of the Scale for Suicide ideation, a systematic interviewing tool developed for the assessment of suicide ideation and risk of future suicidal behavior [1,3]. The BSS inquires about suicidal thoughts and attitudes of subjects toward them. Because the BSS is widely accepted and has strong psychometric properties, the BSS is frequently used in research and clinical practice to assess risk of future suicidal behavior [4].

The role of the Internet in suicide prevention is increasing [5,6]. Online self-help interventions are offered to recover from suicide ideation [7], researchers collect data on suicidal behavior in real time via mobile applications [8,9], and mental health institutions monitor suicidal behavior of patients via online questionnaires [10]. Attrition in online interventions and studies is a well-known problem [11]. To optimize online assessment of patients and thereby limit attrition, there is a need for a shorter and more accurate questionnaire to assess risk of suicidality. Traditional pencil-and-paper mental health questionnaires have a large respondent burden because they require patients to answer questions that do not provide any additional information. In our example, the BSS has 19 items and a score range from 0 to 38. However, a prospective study showed that subjects who scored >2 were 7 times more likely to show future suicidal behavior than those that scored 2 or less [2]. It seems that when assessing risk of future suicidal behavior, if a subject scores >2 there is no need to complete the other items. Computer adaptive testing (CAT) [12] allows us to reduce the number of items in a questionnaire without losing discriminatory validity. Its applicability has been demonstrated in depression [13] and anxiety [14], but not yet in the assessment of the risk of suicidal behavior. Because answering several items on suicidal behavior online can be burdensome for patients, especially at baseline or on intake [15,16], a shorter assessment of suicidal ideation is preferable.

### Computer Adaptive Testing

CAT is possible because of item response theory (IRT) and the wide availability of the Internet. IRT is based on a computerized iterative process that, for each item, regresses the patient’s response on a latent trait score (theta; suicide ideation in our example), the estimated value of which maximizes the likelihood of the patient’s pattern of responses [17]. More concretely, a patient answers an item online and based on the response to that single item, the computer follows an IRT-based algorithm that offers the patient the next most informative item. After the patient’s score has been estimated at the predefined level of precision, no more items are administered. So, only the fewest possible items are offered per patient, resulting in less respondent burden and even more accurate outcomes [17]. Due to these advantages, IRT and CAT are currently being applied in health outcomes research to develop or improve existing measures. For example, The Patient-Reported Outcomes Measurement Information System (PROMIS), a large project funded by the National Institute of Health to develop valid, reliable, and standardized questionnaires to measure patient outcomes [12] relies heavily on IRT and CAT modeling.

### Current Study

The goal of the current study was to investigate whether we can use CAT to shorten the BSS without losing discriminatory validity. We followed the 5 steps of the psychometric analysis plan as used in the PROMIS project [12]. We provided descriptive statistics, evaluated the assumptions for the IRT, fitted an IRT model to our data, tested for item bias, and stimulated a CAT on our data. Because this paper is the first to apply IRT and CAT in the field of suicidology, we explain every step of the process in depth. We have ended this paper with a concrete example of a shortened version of the Dutch version of the Beck Scale for Suicide Ideation (BSS-NL). An overview of the 5 psychometric steps are:

1. Descriptive statistics
2. Testing of assumptions about the IRT model
3. Fitting of the IRT model to the data
4. Evaluating differential item functioning (DIF)
5. Computer adaptive testing

## Methods

### Measurement Procedure

We used the data collected at baseline in the Dutch Professionals in Training to STOP suicide (PITSTOP suicide) study [18]. In the study, mental health professionals were trained in guideline adherence via an e-learning-supported Train-the-Trainer program. Although the intervention was aimed at improving suicide prevention skills of professionals [19], the primary outcome of the study was a change in suicide ideation of patients as measured with the Dutch version of the BSS, the BSS-NL. The BSS was translated into Dutch making use of forward and back translation, and was recently used in a clinical trial study [20]. The preferred mode of data collection among patients was via the routine outcome monitoring (ROM) system, an online system by which data on the effectiveness of treatment in everyday clinical practice are systematically collected [3]. In departments not using ROM, graduate students and/or research assistants used paper-and-pencil questionnaires to collect data. The main *Diagnostic and Statistical Manual of Mental Disorders* (Fourth Edition) *DSM-IV* diagnosis of each patient was assessed at intake via a structured interview by a mental health professional.

All eligible patients were informed about the study and all provided informed consent.

### Software

All analyses were performed in R [21]. Descriptive statistics and principal components analysis (PCA) were obtained via the psych package [22]. The confirmatory factor analysis (CFA) models were estimated using the lavaan package [23]. Graded response models (GRM) were fitted using the latent trait modeling (LTM) package [24]. The mokken package was used to estimate monotonicity [25]. DIF was checked via the lordif package [26]. The CatIRT package was used for the CAT simulation [27].

We followed the 5 steps as used in the PROMIS study as listed in the Introduction.

### Step 1: Descriptive Statistics

Descriptive statistics were described. Cronbach alpha [28] was used to test internal consistency reliability, with .8 as acceptable minimum.

### Step 2: Testing Assumptions About the Item Response Theory Model

Before fitting the IRT model, the basic assumptions for IRT models were tested. The assumptions for IRT are unidimensionality, local independency, and monotonicity [17].

For unidimensionality, we performed a PCA to examine whether a 1-dimensional test explained at least 20% of the variance and whether the ratio of explained variance of the first factor to the second was 4 or higher [29]. Next, we used a CFA to test unidimensionality by using various fit indexes [12]. The residual matrix produced by this single factor CFA was used to test the second assumption, local independence. Correlations >.2 were flagged and considered as possible violations of local independence [12]. Finally, monotonicity was examined by fitting a nonparametric IRT model that resulted in IRT probability curves. Nonmonotonic items with a scalability coefficient <.3 [25] were flagged [12] and described.

### Step 3: Fit an Item Response Theory Model to the Data

There are a great number of different IRT models [30]. For questions with ordered-response categories, the GRM [31] was proposed. Because the BSS has 3 ordered-response options (0, 1, and 2; a higher score represents a higher level of suicide ideation), we fitted a GRM to our data.

**Figure 1:**
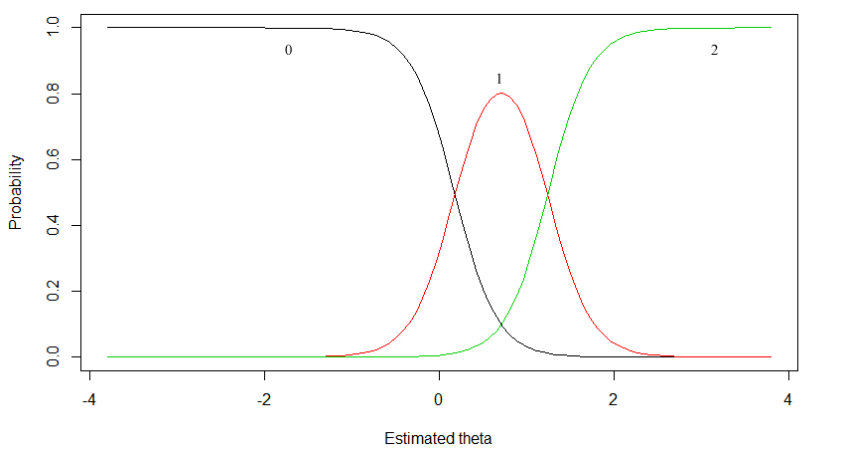
Example of category response curve for item 7 (frequency of thinking about suicide). Number and colors (0: black; 1: red; 2: green) reflect answer options.

As an introduction to the GRM, we provided an example of a GRM for item 7 (frequency of thinking about suicide) of the BSS. Figure 1 shows a function per response category (0, 1, and 2) that corresponds to the chance that a participant chooses that option given a certain score of theta. Because each item of the BSS has 3 response options (0, 1, and 2) per item, 3 curves are presented. The combined chance of all 3 response curves at any certain level of theta is always 1. In other words, in Figure 1, if a patient has a theta of –2, the chance that a patient will choose option 0 is approximately 1. If a patient has a theta of 1.5, he/she has an approximately zero chance of endorsing option 0, 0.65 chance of endorsing option 1, and 0.35 (1–[0+0.65]) of endorsing option 2. Patients that score theta ≥2 will most likely endorse option 2. Every single item is defined by a discrimination parameter (alpha) and 2 location parameters (β_1_ and β_2_). The item parameters of the current example are estimated to be α=4.117, β_1_=0.171, and β_2_=1.243. The discrimination parameter reflects the true difference in theta per item and is comparable to a factor loading. The betas (threshold parameters) indicate the location on the scale of the latent continuum where the item best discriminates among individuals.

To evaluate the fit of the IRT model to the data, category response curves (CRC) for each single item, such as in Figure 1, were plotted.

### Step 4: Evaluation of Differential Item Functioning

Differential item functioning (DIF) exists if patients from 2 groups (eg, men and women) who are equal in terms of the level of theta do not have the same probability of endorsing a test item [32]. Similarly to the PROMIS study, 4 important covariates were considered: gender, age (18-49 years, 50-69 years), education (low level of education/college or advanced degree), and method of administration (computer vs paper and pencil). The IRT item parameters (discrimination and threshold parameters) are assumed to be linear invariant with respect to group membership. Any difference found in CRC then indicates that patients with the same level of theta but from different groups have a different probability of endorsing an item. Items that show DIF at an alpha level of 0.01 were flagged. Because statistical power is dependent on sample size, trivial but nonzero differences are likely to be found to be significant in our large sample. Therefore, we also reported effect sizes to further investigate the magnitude of the DIF. McFadden’s pseudo *R* ^*2*^ <.13 are negligible, and effect sizes between .13 and .26 are moderate [26].

### Step 5: Computer Adaptive Testing

The Package CatIRT performs a post hoc CAT. The IRT parameters obtained in step 3 were used for our CAT simulation unless the DIF analysis suggested using different parameters for subgroups of patients. As a starting point, we set an entry level, which is normally chosen to be 0 [13]. The first item to be selected was the item with the most information at this initial level of suicide ideation. The next item was selected via maximum Fisher information method, related to the theta estimated on basis of the just-selected item and the response to that item. Finally, we determined a stopping rule. As a stopping rule, we used a value of theta that reflects BSS >2 (θ >-1). Our CAT was terminated if the confidence interval surrounding an estimate of theta was fully within 1 of the categories (elevated risk/no elevated risk). We used a confidence interval of 99%. Because questionnaires in mental health care tend to peak at the relative higher levels of the clinical outcome [13,17,33], we also added a second stopping rule to prevent subjects without suicide ideation from having to complete all 19 items. The second stopping rule was use a maximum of 6 items. We compared the classification differences when using none, 1, or 2 stopping rules.

## Results

### Overview

We applied the CAT to the 505 patients within the PITSTOP suicide trial that completed the full 19 items. Initially, data were collected via the ROM. After the start of the study, it appeared difficult for most departments to collect our data via the ROM. In total, only 43% (217/505) of the data was collected using the ROM. As an alternative, research assistants and clinicians were instructed to complete the questionnaire via paper and pencil. Of the 505 patients, 93 (18.4%) patients had a total BSS score of 0, and 254 (50.3%) had a score <8; 128 (25.3%) had depression as their primary diagnosis and 50 (9.9%) had a personality disorder. Mean age was 42 (SD 9.2) years. At baseline, 183 of 505 (36.2%) patients stated they had attempted suicide at least once.

### Step 1: Descriptives

The overall Cronbach alpha was .94. Average score on the BSS was 10.4 (SD 9.4). As Table 1 shows, removing 1 item did not lead to a substantial improvement of the internal consistency. The item-rest or remainder correlations (*R*_rest_) were also satisfactory.

**Table 1:** Descriptive statistics of the single items of the BSS-NL.

Single item content	Category			Mean (SD)	Cronbach α	*R*_rest_
	0	1	2			
1. Wish to live	242	195	68	0.66 (0.79)	0.94	.67
2. Wish to die	199	196	110	0.82 (0.70)	0.93	.74
3. Reasons living/dying	278	157	70	0.59 (0.84)	0.93	.69
4. Desire to kill oneself	246	171	88	0.69 (0.76)	0.93	.80
5. Passive suicidal desire	248	174	83	0.67 (0.77)	0.93	.63
6. Duration of suicide ideation	319	113	73	0.51 (0.90)	0.93	.76
7. Frequency of thinking about suicide	310	160	35	0.46 (0.96)	0.93	.80
8. Acceptance of idea of suicide	285	153	67	0.57 (0.85)	0.93	.73
9. Control over suicide action	344	140	21	0.36 (1.08)	0.93	.68
10. Reasons for not committing suicide	314	138	53	0.48 (0.93)	0.93	.70
11. Reasons for wanting to commit suicide	223	47	235	1.02 (0.67)	0.93	.48
12. Specific plan to commit suicide	318	118	69	0.51 (0.91)	0.93	.71
13. Access to suicide method	335	31	139	0.61 (0.82)	0.93	.60
14. Courage/ability to commit suicide	279	152	74	0.59 (0.83)	0.93	.74
15. Expectation to commit suicide	318	153	34	0.44 (0.98)	0.93	.76
16. Preparations for suicide	391	89	25	0.28 (1.19)	0.93	.63
17. Writing of suicide note	394	72	39	0.30 (1.16)	0.93	.49
18. Final acts in anticipation of death	354	106	45	0.39 (1.04)	0.93	.37
19. Conceal ideation	308	123	74	0.54 (0.88)	0.93	.47

### Step 2: Testing of Assumptions of the Item Response Theory Model

When fitting a 1-factor PCA, we found that 50% of the proportional variance was explained by the first factor. The ratio between a 1- and a 2-factor model indicated that the first factor model explained 14 times more variance than the second factor. When fitting a confirmatory analysis we found a comparative fit index of 0.999, a Tucker-Lewis index of 0.989, a root-mean-square error of approximation of 0.045 (90% CI 0.038-0.053), and a standardized root-mean-square residual of 0.059.

### Step 3: Fitting of a Graded Response Model

#### Overview

Table 2 shows that all 19 items had an alpha higher than 1. Item 7 (frequency of thinking about suicide) seems to discriminate best between patients with a higher or lower level of suicidal ideation, as indicated by the high alpha of 4.117.

**Table 2:** Graded response model parameters for the Dutch version of the Beck Scale for Suicide Ideation (BSS-NL).

Item and content	Parameter		
	α	β_1_	β_2_
1. Wish to live	2.366	0.029	0.556
2. Wish to die	3.197	–0.159	0.180
3. Reasons living/dying	3.036	0.034	0.937
4. Desire to kill oneself	4.082	–0.123	0.691
5. Passive suicidal desire	2.270	–0.188	0.898
6. Duration of suicide ideation	3.434	0.276	1.054
7. Frequency of thinking about suicide	4.117	0.171	1.243
8. Acceptance of idea of suicide	3.437	0.071	0.985
9. Control over suicide action	3.165	0.386	1.587
10. Reasons for not committing suicide	3.048	0.263	1.258
11. Reasons for wanting to commit suicide	1.557	0.001	0.100
12. Specific plan to commit suicide	3.003	0.305	1.122
13. Access to suicide method	2.479	0.550	0.616
14. Courage and ability to commit suicide	3.780	0.045	0.932
15. Expectation to commit suicide	3.825	0.232	1.369
16. Preparations for suicide	2.532	0.754	1.815
17. Writing of suicide note	1.786	1.016	1.952
18. Final acts in anticipation of death	1.098	0.887	2.414
19. Hide, conceal, or lie about suicide ideation	1.436	0.334	1.466

#### Category Response Curves

Of the 19 items, 17 showed CRC plots as expected. Items 11 (reasons for wanting to commit suicide) and 13 (access to suicide method) showed CRCs that warranted extra inspection. Table 3 shows the mean overall theta of participants per response option for 3 different items: item 7, which had a good CRC, and for items 11 and 13, which showed unsatisfactory CRCs. For items 11 and 13, the difference in mean theta for responses 1 and 2 was small and their confidence intervals overlapped, indicating that a higher score on 1 of these items does not necessarily reflect a higher level of suicidal ideation.

**Table 3:** The mean theta of patients that endorsed response option 0, 1, and 3 for items 7, 11, and 13.

Item and response		Mean θ	95% CI
**7. Frequency of thinking about suicide**			
	0	–1.7	–2.0, –1.4
	1	1.4	1.3, 1.5
	2	2.6	2.4, 2.8
**11. Reasons for wanting to commit suicide**			
	0	–2.3	–2.6, –1.9
	1	0.9	0.6, 1.2
	2	1.0	0.8, 1.1
**13. Access to suicide method**			
	0	–1.5	–1.7, –1.2
	1	1.3	1.1, 1.6
	2	1.6	1.4, 1.7

### Step 4: Differential Item Functioning

No items were flagged for DIF when analyzing differences in gender, age, or education. When analyzing the effect for the administration method, 7 items were flagged. However, *R* ^*2*^ were all <.13.

### Step 5: Computer Adaptive Testing

**Figure 2:**
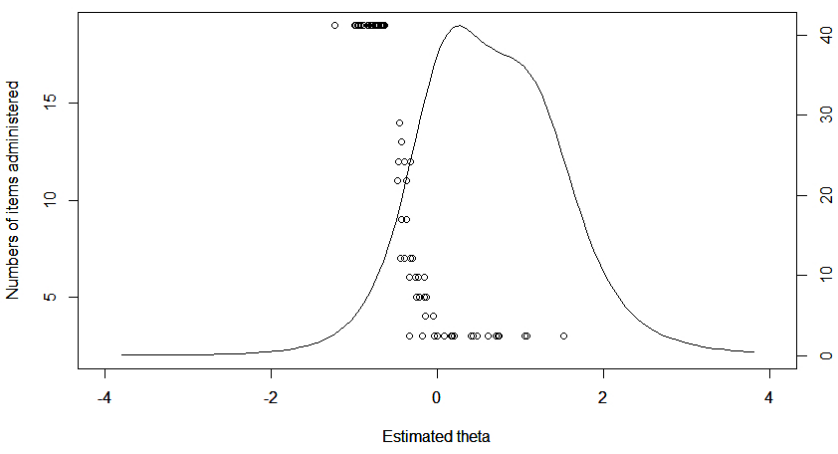
Relationship between level of theta and the number of administered items under stop rule 1. The curve represents test information as a function of theta.

When administrating all 19 items, 345 patients were classified as being at risk (Table 4). When allowing the number of items to vary between 3 and 19, CAT simulations showed that, on average, 10 items were sufficient to meet the same classification as the first model. For a large number of patients with a low trait of suicidal ideation, all items were exhausted before the stopping rule was met (Figure 2). When using a maximum of 6 items, 336 instead of 345 patients were classified as having an elevated risk (Table 4).

**Table 4:** Classification of risk for several stopping rules for Beck Scale for Suicide Ideation scores >2.

Stopping rule		Mean (SD)	Number of patients with low risk of future suicidal behavior	Number of patients with elevated risk of future suicidal behavior
Min items	Max items			
19	19	19 (0)	160	345
3	19	9.7 (7.7)	162	343
3	6	4.2 (1.4)	169	336

## Discussion

### Principal Findings

Our simulation showed that an IRT model can be fitted to the BSS-NL and that CAT can successfully be applied to reduce the length of the BSS-NL when assessing risk of future suicidal behavior. PCA and confirmatory factor analysis found the scale to be highly unidimensional. No local independence or violation of monotonicity was found. Therefore, all assumptions for IRT modeling were met. For 17 of 19 items, IRT parameters were satisfactory indicating that most items are well suited to provide differential information on a patient’s level of suicidal ideation. When using CAT with a maximum of 6 items, only 9 of 505 (1.7%) patients were classified in a different category when compared to the classification under all 19 items. Importantly, this simulation demonstrated that CAT makes it possible to administer only 4 items, on average, instead of the full 19 without losing discriminatory validity.

### Improvement of the Items 11 and 13

We found items 11 (reasons for wanting to commit suicide) and 13 (access to suicide method) to show unsatisfactory item parameters. Further inspection revealed that, for both items, patients with comparable levels of suicide ideation were equally likely to endorse either option 1 or 2. For example, consider item 13. Our data showed that patients with low suicidal trait were more likely to endorse option 0 (have no access to means) and patients with higher levels of suicidality were equally likely to endorse option 1 (it takes time to find means) or 2 (I have access to means). Due to this overlap, patients with the same level of suicidal ideation might end up with different summed total scores. Therefore, when using the full-scale version of the BSS, we advise rephrasing the response options of both items, offering them as dichotomous items or excluding them.

### Strengths and Limitations

Because this is a simulation study, real-time CAT studies are needed to determine the most accurate item parameters. Few clinical studies have implemented CAT in real time, but those studies that did showed a good comparison with simulation studies (eg, [34]). Next, it is necessary to compare the parameters of the current study with, for example, data collected with the original English-language version of the BSS. For our simulation, we used a fixed theta as cut-off score instead of the established BSS score >2. Future prospective studies must examine the most plausible theta cut-off to predict elevated risk of suicidal behavior. Also, we had no long-term follow-up data on whether patients actually engaged in any suicidal behavior after the assessment. Therefore, we were not able to compare the predictive validity of the CAT with the predictive validity of the full test. An additional limitation of CAT approaches might be that CAT data would not be comparable to normative data. By standardizing outcomes as done in meta-analysis [35], scores assessing the same outcome but measured in a variety of ways can still be compared.

With the BSS-NL, it seems to be difficult to investigate small differences in patients with a low suicidal trait. This has been found more often in mental health assessments [13,17,33]. A hybrid CAT approach, such as the 2-stage semiadaptive testing strategy recommended by Choi et al [35], might also be appropriate and result in even more accurate classification.

Finally, although wireless Internet and reliable hardware are widely available, the current state of ICT in (Dutch) mental health care reduces the feasibility of large-scale CAT implementation. Even for our normal (non-CAT) assessments, many of the research assistants within our study had to resort to paper-and-pencil testing because computerized testing was technically not possible. Obviously, this precludes CAT.

Strengths of this study pertain to its large sample size; 505 patients from various psychiatric departments completed the BSS. Therefore, the external validity of the findings is considerable. Another strength of the current paper is the application of modern psychometric techniques within the field of clinical psychology/psychiatry. For several reasons, such as lack of interest in new techniques and insufficient mathematical training, the integration of new techniques in psychology/psychiatry has been suboptimal at least [36]. By thoroughly explaining every step of our analysis and by focusing on the actual application of IRT and CAT in the clinical field, this paper hopes to stimulate the use of contemporary psychometric techniques.

### Concrete Example of the Computer Adaptive Testing

**Figure 3:**
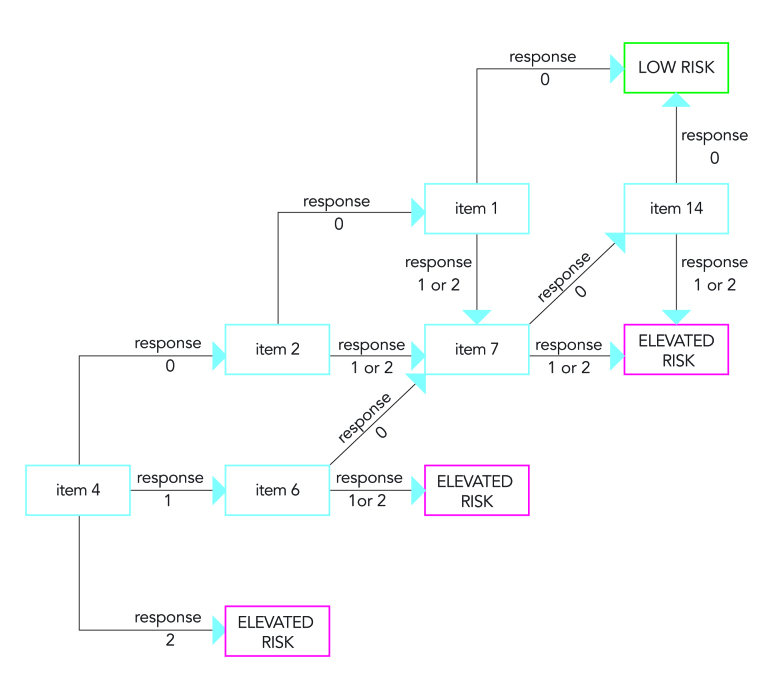
Concrete example of a result of a CAT simulation.

As stated previously, due to the mathematical and computational modeling, IRT and CAT can be a bit daunting for clinicians and applied scientists. Therefore, we provide a concrete example of our last CAT simulation (theta bound=–1, max items=6) (Figure 3). In our simulation, all patients started with item 4. Based on the answer to item 4, either item 2 or item 6 is selected, or the person is at an elevated risk (if the participant answers with response 2). For example, if a patient choose response 1 for item 4 (“I have a weak desire to kill myself”), the next most informative item would be item 6 (“length of periods of thinking about killing oneself”). If a patient answers that item with either moderate or long periods (responses 1 or 2), they would be categorized to be at high risk. If a patient selects response 0 (very brief periods), the next item would be item 7 (frequency of suicidal thoughts). Following this algorithm, a high-risk patient needs only 1 or 2 items to be classified as having an elevated risk.

### Conclusions

One of the main advantages of CAT is the reduction of respondent burden. Answering items on suicidal behavior online can be difficult for patients, resulting in a high dropout rate. Because attrition is a well-known problem in eHealth, reducing response burden of online assessment of suicidal behavior is important. It should be noted that our CAT simulation showed that the number of items can be considerably reduced when using the BSS-NL to assess elevated risk of suicidal behavior. Our simulation showed that 4 items, on average, were sufficient. Obviously, for CAT to be widely accepted and implemented, many more (prospective) studies should be done and ICT within mental health or research settings should be drastically improved. However, considering the need for rapid yet accurate online assessment of suicide risk in both clinical and research practice, we argue that IRT and CAT are likely to play important roles in the development of better measurement methods for the assessment of risk of suicidal behavior.

## Abbreviations

BSS: Beck Scale for Suicide Ideation
BSS-NL: Dutch version of the BSS
CAT: computer adaptive testing
DIF: differential item functioning
IRT: item response modeling
PCA: principal component analysis
PITSTOP suicide: Professionals in Training to STOP suicide
PROMIS: The Patient-Reported Outcomes Measurement Information System
Rrest: item-rest correlation
